# Case report: Giant cystic ileal gastrointestinal stromal tumor with an atypical intratumoral abscess

**DOI:** 10.3389/fsurg.2022.1056831

**Published:** 2023-01-06

**Authors:** Linguang Chen, Jiannan Gu, Xuejun Zhang, Aijun Yu

**Affiliations:** Department of the First General Surgery, Affiliated Hospital of Chengde Medical University, Chengde, China

**Keywords:** gastrointestinal stromal tumor, cystic neoplasm, intratumoral abscess, small intestine, case report

## Abstract

**Background:**

Gastrointestinal stromal tumors (GISTs) are typically solid, sometimes with small cystic areas, but rarely manifest as predominantly cystic neoplasms. In addition, cystic intestinal GISTs with intratumoral abscess formation are rare.

**Case presentation:**

We present the case of a 49-year-old male patient with a history of frequent and urgent urination for 2 weeks. Radiologic studies revealed a large cystic mass in the lower abdomen. The patient underwent abdominal laparotomy, which revealed a large cystic mass arising from the distal ileum invading the sigmoid mesocolon and apex vesicae. Partial resection of the ileum along with the tumor and the adjacent bladder was performed. Macroscopic examination revealed that the cystic mass contained a large amount of foul-smelling pus and a tumor-bowel fistula. The final pathology revealed an abdominal stromal tumor. Postoperative recovery was uneventful, and adjuvant imatinib mesylate 400 mg was administered daily. No tumor recurrence or metastasis was observed during the 9-month follow-up period.

**Conclusion:**

Fingings of a cystic tumor in the abdomen should raise concern for cystic GISTs. This case report reviews a rare presentation of an ileal giant cystic GIST with atypical intratumoral abscess formation. Complete surgical resection and adjuvant imatinib is still the mainstay treatment for GISTs.

## Introduction

Gastrointestinal stromal tumors (GISTs) are the most common mesenchymal tumors of the gastrointestinal tract, accounting for 0.1%–3% of all gastrointestinal tumors (1), with an estimated annual incidence of 10–15 cases per million people (2). Most GISTs originate from the stomach (60%), with the small intestine being the second most common location. Small intestinal GISTs comprise approximately 30% of all GISTs (3), and most are located in the jejunum, followed by the duodenum and ileum (4). GISTs are typically solid tumors that rarely present with predominant cystic changes. Therefore, cystic intestinal GISTs with intratumoral abscess formation are rare. Herein, we report a case of cystic ileal GIST that presented as a large abdominal cystic tumor preoperatively. In addition, a large amount of foul-smelling pus was observed within the tumor intraoperatively. A rapid frozen pathologic examination showed a mesenchymal tumor composed of spindle cells, and a postoperative pathological examination confirmed the diagnosis of GIST. Since a majority of previously reported cases of GISTs with an intratumoral abscess had findings of fever and significant abdominal pain, examination for an internal air-fluid level through imaging followed by emergency surgery should be performed (5–7). However, the present case had no such typical features; therefore, it was an “atypical abscess.” To the best of our knowledge, this is the first report of an ileal giant cystic GIST (cGIST) with an atypical intratumoral abscess. This study was reported in agreement with principles of the CARE guidelines (8).

## Case report

**Figure 1:**
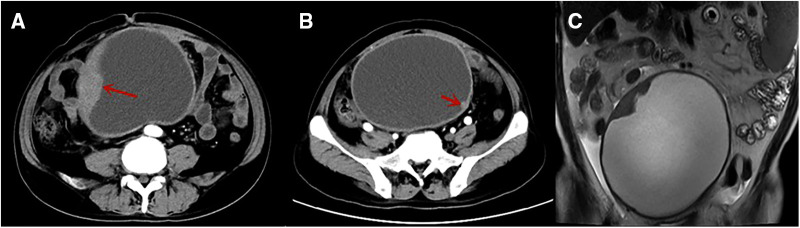
Ct scan showing a large cystic tumor in the lower abdomen with an unevenly thickened cystic wall, the solid component of the tumor (red arrow, **A**), and mural nodules (red arrow, **B**) with enhancement in the arterial phase. MRI revealed a large cystic tumor with an internal fluid signal (**C**). CT, computed tomography; MRI, magnetic resonance imaging.

A 49-year-old man was admitted to our hospital with a history of frequent and urgent urination for 45 the past 2 weeks. He did not have abdominal pain, fever, dysuria, or hematuria. His medical history was unremarkable except for hypertension. Physical examination revealed distention of the lower abdomen. A large, painless, immobile, and hard mass was palpable, having an estimated diameter of 17 cm. Laboratory testing revealed the following: C-reactive protein, 61.50 mg/L (normal range, 0–8 mg/L); white blood cell count, 14.94 × 109/L (normal range, 3.5–9.5 × 109/L); neutrophilic granulocytes, 80.8% (normal range, 40%–75%); hemoglobin, 101 g/L (normal range, 130–175 g/L); procalcitonin, 0.25 ng/ml (normal range, 0–0.05 ng/ml); interleukin-6, 43.49 pg/ml (normal range, 0–10 pg/ml); prothrombin time, 14.3 s (normal range, 9–13 s) and D-dimer, 1.60 ug/ml (normal range, 0–0.55 ug/ml). Routine urine tests, liver function tests, and tumor markers (AFP, CEA, CA 19-9) were all within the normal ranges. Ultrasonography revealed a large cystic mass, measuring approximately 17 cm in diameter, in the lower abdomen in front of the abdominal aorta and iliac vessels. Contrast-enhanced computed tomography (CT) revealed an abdominal cystic tumor with an unevenly thickened wall and mural nodules; air-fluid levels were not observed within the tumor. Enhancement was noted in the cyst wall and mural nodules during the arterial phase (Figures 1A,B). Magnetic resonance imaging (MRI) revealed a large cystic tumor with an internal fluid signal (Figure 1C). The nature and origin of the tumor could not be diagnosed preoperatively; therefore, a diagnostic laparotomy was performed through a midline abdominal incision. The procedure revealed a large cystic tumor arising from the distal ileum invading the sigmoid mesocolon and apex vesicae. Partial resection of the ileum along with the tumor and adjacent bladder was performed. The tumor was sent for frozen sectioning, revealing proliferated spindle-shaped tumor cells (Figure 3A). Anastomosis of the ileal was then performed using staples, side-to-side, without tumor rupture. Macroscopic examination revealed that the excised cystic mass measured 17 cm in the largest dimension and contained a large amount of foul-smelling pus.

**Figure 2:**
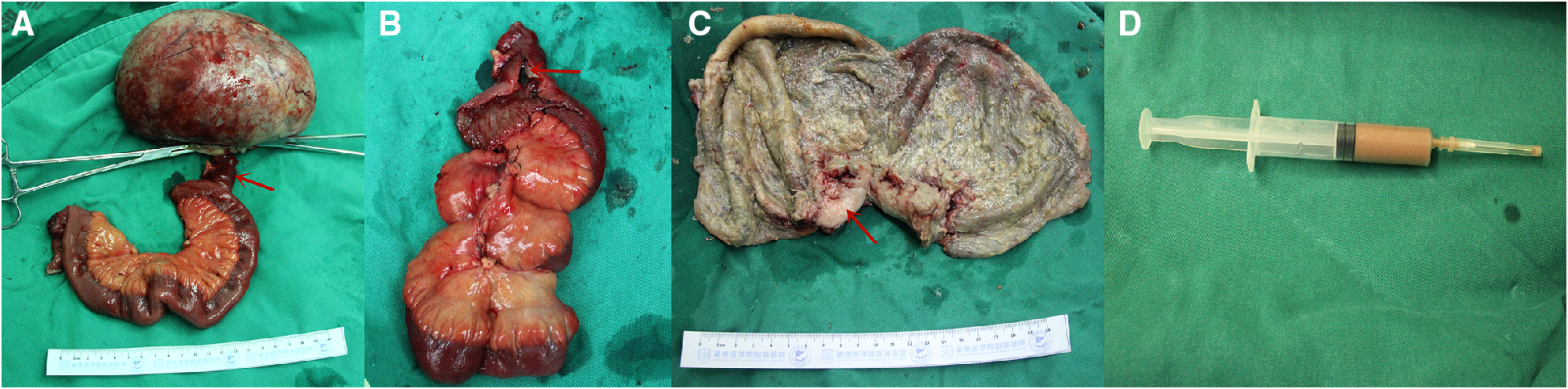
Intraoperative photograph showing a large cystic tumor arising from the ileum (**A**), communicating with the ileum *via* a small intestinal fistula (red arrow, **A,B**). The red arrow indicates the thickening of the cystic wall, which was the solid component of the tumor (red arrow, **C**). The last photo shows pus within the tumor (**D**).

**Figure 3:**
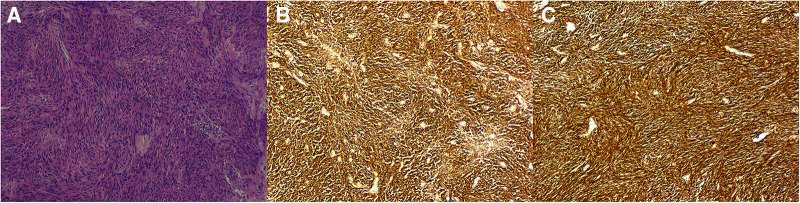
Hematoxylin and eosin staining showing a large number of spindle cells (10 × 10, **A**). Immunohistochemical staining showing tumor cells positive for c-kit (**B**) and DOG-1 (**C**).

Further examination revealed that the tumor communicated with the intestinal lumen *via* a mucosal fistula (Figure 2). The pus culture test result was positive for Salmonella enteritidis. Immunohistochemical staining showed that the tumor cells were positive for c-kit, DOG-1, CD34, and vimentin but negative for S-100 (Figures 3B,C). The Ki-67 index was approximately 6%. Mitotic count was less than 5/50 high-power fields. The resection margins were negative, and there was no lymph node metastasis. Gene analyses revealed the presence of a c-kit exon 11 mutations. The final diagnosis was high-risk GIST originating from the ileum. Postoperative recovery was uneventful, and there were no postoperative complications. The urine tube was removed 1 week after surgery, and the patient was discharged on the 13th postoperative day. Adjuvant imatinib mesylate (400 mg daily for 3 years) was administered. The patient did well during the 9-month follow-up visit without tumor recurrence or metastasis.

## Discussion

GISTs typically present as regular solid exoplastic masses, primarily found within the gastrointestinal tract and occasionally outside. GISTs rarely present as predominantly cystic tumors; in case of this rare occurrence, some researchers define them as cGISTs; if the proportion of cystic components is larger than 75% and the cyst wall is relatively regular according to the corresponding gross examination or preoperative radiological reports. The cGISTs should be considered a specific subtype of GISTs with relatively indolent behaviors and favorable prognoses, although similar to solid GISTs in terms of morphological and immunohistochemical features (9). The cause of cGISTs is related to degeneration, necrosis, and bleeding, although the exact mechanism remains unknown (9, 10). In addition, imatinib treatment can induce cystic changes (11).

Small bowel GISTs (SB GISTs) are the second most common type of GISTs in the digestive tract, with 5-, 10-, and 20-year disease-specific survival rates of 84.4, 71.2, and 54.2%, respectively (12). A retrospective study showed that patients with cGISTs had a 9:11 male-to-female ratio, with a mean age of 61 years (9). As SB GISTs often show an exophytic growth pattern and high activity, they tend to be asymptomatic in the early stages. Abdominal pain and gastrointestinal bleeding were the most common symptoms of cGISTs (9). Other rare presentations include obstruction, abdominal hemorrhage, tumor rupture, and peritonitis, which usually require emergency surgical intervention and are more common in the small intestine than in gastric GISTs (13). GIST-related fistulas and intratumoral abscesses are rare. Previous studies have suggested that the mechanism of intratumoral abscess formation comprises enteric bacteria entering the tumor cavity through the tumor-small intestinal fistula, which occurs due to GISTs' propensity to cause mucosal ulceration or defects, and eventually develops into an intratumoral abscess (5, 6, 14). There have been case reports of bacteremia and pyogenic liver abscesses resulting from enteric bacteria entering systemic circulation through the portal vein (14, 15). However, the present case had no typical features, such as a previously reported abdominal abscess or hyperpyrexia, severe abdominal pain, air-fluid level on imaging, and other indications for emergency surgery (5–7). He only had a mild elevation of inflammatory markers; therefore, it was referred to as an “atypical abscess.” We speculate that the patient was in the early stage of the disease without bacteremia or infection due to gas-producing bacteria.

CT or MRI is of great significance in diagnosing cGISTs. cGISTs usually demonstrated as an exophytic, well-defined, low-density mass with peripheral enhancement on contrast imaging (9).Tumor calcification was observed in 22% of patients, and tumor-related complications, such as tumor-bowel fistula, bowel obstruction, and intraperitoneal rupture, were observed in 32% of patients (16). However, CT or MRI is insufficient for preoperative diagnosis, especially for cGISTs. In addition, it is difficult to differentiate it from other cystic diseases such as duplication cysts, mucin-producing tumors, pancreatic pseudocysts, and cystic lymphangiomas. ^18^F-fluorodeoxyglucose positron emission tomography (^18^FDG-PET) can be helpful in differentiating malignancy and ruling out metastatic disease as well as monitoring response to molecularly targeted therapy. Moreover, ^18^FDG-PET is more sensitive for the assessment of early therapy response than morphologic imaging modalities (17).

A recent systematic review and a retrospective cohort study showed diagnostic biopsies are safe procedures, with a very low risk of needle tract seeding and without an increase in local recurrence rates (18, 19). Endoscopic ultrasound-guided fine-needle aspiration (EUS-FNA) may provide an opportunity for preoperative pathological diagnosis of gastric cGISTs (20, 21). However, the safety for SB cGISTs needed to be further studied, especially for giant cystic lesions.

Histological examinations, immunohistochemical features and mutational analysis are useful for the final diagnosis of GISTs. Histologically, the most common cell morphology was the spindle cell type (86%), followed by the epithelial type (5%), and mixed type (9%). Immunohistochemical tests indicated that KIT, CD34, SMA, S-100, and desmin expression rates were 98%, 40%, 34%, 14%, and 0.2%, respectively (22). Mutational analysis showed that KIT mutations are present in 75% of GISTs, whereas 10% have platelet-derived growth factor receptor alpha mutations (23).

Surgical resection and imatinib are the most effective treatment for primary GISTs. For cGISTs, high attention should be paid intraoperatively to avoid abdominal dissemination due to tumor rupture. Partial resection of the ileum along with the tumor is the standard procedure for ileal cGISTs and lymphadenectomy should not be routinely performed if there is no evidence of lymph node metastasis. It is well accepted patients with high-risk features had prolonged overall survival (OS) with adjuvant imatinib (24). However, a previous study showed that oncologists tend to frequently underestimate the risk of GIST recurrence after initial tumor resection, especially in patients with intermediate tumor size (6–10 cm), intermediate-level mitotic count (6–10/50 HPF), and non-gastric origin, which affects the duration of postoperative adjuvant therapy and recurrence-free survival (25). In addition, preoperative imatinib therapy can effectively prevent tumor rupture and reduce post-surgical complication as well as improve prognosis for large GIST patients (26, 27). Therefore, for resectable cases of GISTs, accurate assessment of post-resection risk, standardized adjuvant/neoadjuvant therapy, and follow-up are crucial to improve prognosis and reduce postoperative recurrence.

## Conclusion

We present the case of a large cGIST located in the ileum with an intratumoral abscess diagnosed through histopathology after surgical excision. For intraperitoneal cystic lesions, especially those suspected to originate from the gut, the possibility of a GIST should be considered before surgery. We have shared our clinical experience to help guide the management of similar cases.

## Data Availability

The original contributions presented in the study are included in the article/Supplementary Material, further inquiries can be directed to the corresponding author/s.
